# Multi-tissue transcriptome analysis to identify candidate genes associated with weight regulation in Hanwoo cattle

**DOI:** 10.3389/fgene.2023.1304638

**Published:** 2024-01-09

**Authors:** Subin Jang, Sunsik Jang, Jaemin Kim, Woncheoul Park

**Affiliations:** ^1^ Division of Applied Life Science (BK21), Gyeongsang National University, Jinju, Republic of Korea; ^2^ Institute of Agriculture and Life Sciences, Gyeongsang National University, Jinju, Republic of Korea; ^3^ Hanwoo Research Institute, National Institute of Animal Science, Rural Development Administration, Pyeongchang, Republic of Korea; ^4^ Animal Genomics and Bioinformatics Division, National Institute of Animal Science, Rural Development Administration, Wanju, Republic of Korea

**Keywords:** Hanwoo, body weight, eQTL analysis, differentially expressed genes, transcriptome

## Abstract

While genetic markers related to meat production traits have been identified in many other cattle breeds, research on weight in Hanwoo cattle (Korean native cattle) is still insufficient. In this study, we performed expression quantitative trait loci (eQTL) analysis and differential gene expression analysis to detect candidate genes influencing the weight characteristics of 32 castrated Hanwoo cattle across 22 tissues and, we identified variants that affect gene expression levels. In total, we identified a total of 3,298 differentially expressed genes, among which we discovered key genes such as *UBD*, *RGS2*, *FASN*, and *SCD* that have functions related to adipogenesis, body weight, obesity, and lipid metabolism. Gene-set enrichment analysis revealed that candidate genes in adipose tissue are involved in metabolic pathways linked to obesity-related traits, adipose metabolism, and lipid metabolism. Additionally, we found that decreased expression of *TRIM31* contributes to weight gain which can be explained by the associated candidate *cis*-eQTL genotypes for *TRIM31* and their effect on differential gene expression between the lower and higher weight groups. Our findings revealed candidate genes associated with the weight of Hanwoo cattle and perhaps can provide comprehensive insights into the association of weight with various tissues beyond adipose tissue and muscle, indicating the potential for expanding the focus of livestock trait research.

## Introduction

Hanwoo cattle (Korean native cattle) is a breed of cattle indigenous to Korea that was previously used for agricultural, transportation and religious purposes but later evolved into beef cattle and remains one of the country’s most important food sources to this day (Lee et al., 2014). Hanwoo cattle are recognized for their high fertility, but their slow growth rate hinders their meat production capability (Choi et al., 2019). For efficient meat production of beef cattle, it is important to maximize their weight, which is an economic trait (Fink et al., 2017). Livestock weight is economically important because it indicates livestock ability, a standard for determining livestock rations and selling prices, and is also used as a trait to evaluate livestock breeding value (Wangchuk et al., 2018). To this end, a significant amount of genetic research has focused on elucidating the genetic determinants of body weight and related traits in cattle and other livestock species (Littlejohn et al., 2012).

Genetic studies have progressively shed light on the complex underpinnings of body weight traits in cattle. For instance, Duan et al. (2021) pinpointed several single nucleotide polymorphisms (SNPs) in Chinese Simmental cattle that correlate with body weight, revealing genes that govern growth and development. Similarly, Naserkheil et al. (2020) conducted a GWAS that identified genes in Hanwoo cattle associated with metabolic processes and growth, highlighting the genetic complexity of these traits. Furthermore, Liu et al. (2018) provided insight into the role of lipid metabolism by analyzing differentially expressed genes (DEGs) in the subcutaneous adipose tissue of Lilu cattle, underscoring the multifaceted nature of weight regulation at the genetic level.

While these studies offer valuable information, they primarily rely on GWAS for gene identification, and little attention has been given to integrating transcriptomic data with expression quantitative trait loci (eQTL) mapping to gain a more holistic understanding of weight traits. Recognizing this gap, our study harnesses RNA-seq technology to profile gene expression across Hanwoo cattle tissues, integrating eQTL analysis to elucidate the genetic mechanisms influencing weight. The eQTL analysis is crucial in elucidating the significant association between gene expression levels and genetic polymorphisms, providing a profile that highlights the unique biological significance (Peng et al., 2018; Cai et al., 2023). This comprehensive approach aims to build on the existing genetic framework, adding depth to our understanding of how genetic variations contribute to phenotypic expressions related to body weight in Hanwoo cattle.

In recent studies on weight and body composition traits of Chinese Lilu cattle and common beef breeds in the United States (such as angus, beefmaster, brahman, etc.), identified significant candidate genes have been reported to be involved in lipid metabolism pathways (Liu et al., 2018; Lindholm-Perry et al., 2020). Lipid metabolism, which contributes to the characteristics of body weight, encompasses a range of biochemical pathways including fat synthesis, lipolysis, lipid transport, and oxidation (Muradian et al., 2015). These processes occur not only in adipose tissue but also in various other tissues such as the brain, liver, and muscles (Adibhatla and Hatcher, 2008; Yang et al., 2013; Zhang et al., 2022). Therefore, the aim of this study was to perform expression quantitative trait loci (eQTL) analysis and differential gene expression analysis in different tissues to detect candidate genes influencing weight in Hanwoo cattle.

## Materials and methods

### Experimental overview and sample collection

To identify expression quantitative trait loci (eQTL) in Hanwoo cattle, 32 animals from the same farm were provided by the Hanwoo Cattle Research Institute, National Institute of Animal Science, South Korea. The age (Mean ± Sd, 15.6 ± 5.5) and body weight (Mean ± Sd, 388.9 ± 115.6) of the 32 samples were measured at the time of slaughter (Supplementary Table S1). The 22 tissues collected for RNA-sequencing are as follows: abdominal fat (ABF), abomasum (ABO), back fat (BFT), blood (BLO), cecum (CEC), colon (COL), duodenum (DUO), heart (HEA), ileum (ILE), jejunum (JEJ), kidney (KID), kidney fat (KIF), liver (LIV), sirloin (LOM), lung (LUN), omasum (OMA), rectum (REC), reticulum (RET), round (RMP), rumen (RUM), spleen (SPL) and tenderloin (TEN). Three of 32 individuals (Sample IDs: 192018, 192032, 202012) included missing tissue samples. Information about the tissues collected per individual is provided in Supplementary Table S2. Ethics approval was obtained from the National Institute of Animal Science (approval no: NIAS20201979).

### RNA isolation and sequencing

Tissue samples harvested from 32 castrated Hanwoo cattle were processed for RNA preparation using two distinct methods. The first method involved RNA extraction following the Trizol Beating RLT Dnase column protocol, utilizing the QIAamp 96 Viral RNA Kit in conjunction with QIAzol Lysis Reagent. The second method extracted RNA based on the Trizol beating isopropanol column DNase tissue RNA protocol, employing the QIAamp DNA Mini Kit and QIAzol Lysis Reagent. Additionally, for the blood samples, RNA was extracted by referencing the 900 µL Trizol Isopropanol column protocol, using a combination of QIAzol_3X and QIAzol Lysis Reagent. RNA concentration was checked using a NanoDrop ND-1000 spectrophotometer (NanoDrop Technologies, United States). RNA extracted from tissues and blood was all subjected to RNA QC using the TapeStation RNA Screen Tape, and the criteria were Concentrations (total amount) > 0.5 (ug), RINs value > 6, and rRNA ratio > 1. The quality and the integrity of the RNA was assessed using a bioanalyzer (Agilent, Santa Clara, United States) and only samples with a RIN value greater than 8.0 was used for cDNA library construction. Individual libraries were generated using Illumina TruSeq™ RNA Sample Preparation Kit (Illumina, San Diego, CA, United States). All samples were sequenced on the Illumina NovaSeq 6000 sequencer, generating 100bp paired-end reads at a sequencing depth of 6 Gb. Sequencing for all samples was conducted across separate lanes as per the workflow schedule, rather than being performed on a single lane. The goal was to produce data of at least 60 million reads for each sample. The raw reads were freely deposited at the National Center for Biotechnology Information (NCBI) Sequence Read Archive (SRA) database under accession number E-MTAB-13398.

### RNA-seq data production and RNA SNP calling

We sought to obtain quantified expression values transcripts per million (TPM) to compare gene expression levels and for use in eQTL analysis. RNA-seq data of the 22 tissues sourced from 32 samples were quality-checked with FastQC (version 0.11.9) (Brown et al., 2017), and low-quality reads were filtered through the Trimmomatic (version 0.39) process (Bolger et al., 2014). Expression levels were quantified using the rsem-calculate-expression function of the RNA-seq by expectation maximization (RSEM) software (version 1.3.1) (Li and Dewey, 2011), generating TPM values for each of the 22 tissues across the 32 samples. The ARS-UCD1.2 of cattle was used as the reference genome.

To acquire single nucleotide polymorphisms (SNP) information requisite for principal component analysis (PCA) and eQTL analysis, SNP data was derived from RNA-seq datasets. RNA-seq data from 32 samples of BLO tissue were subjected to SNP calling using genome analysis toolkit (GATK) (version 4.1.4.0) (DePristo et al., 2011) adhering to the best practice guidelines (https://gatk.broadinstitute.org/hc/en-us/articles/360035531192-RNAseq-short-variant-discovery-SNPs-Indels). First, after the previously mentioned Trimmomatic process, we mapped the reference using the spliced transcripts alignment to a reference (STAR) (version 0.11.9) tool (Dobin et al., 2013) and removed duplicates using the MarkDuplicatesSpark tool in GATK. After going through the SplitNCigarReads process of the GATK and base quality recalibration, variant calling was performed using the HaplotypeCaller tool. To enhance the accuracy of RNA SNP variants, we treated genotypes with a genotype quality (GQ) less than 20 and a read depth (DP) less than 5 as missing. SNPs exceeding a 10% missing rate (-geno 0.1) were filtered. We then excluded the sex chromosomes and indels, focusing our analysis solely on autosomal SNPs.

### Study design

**FIGURE 1:**
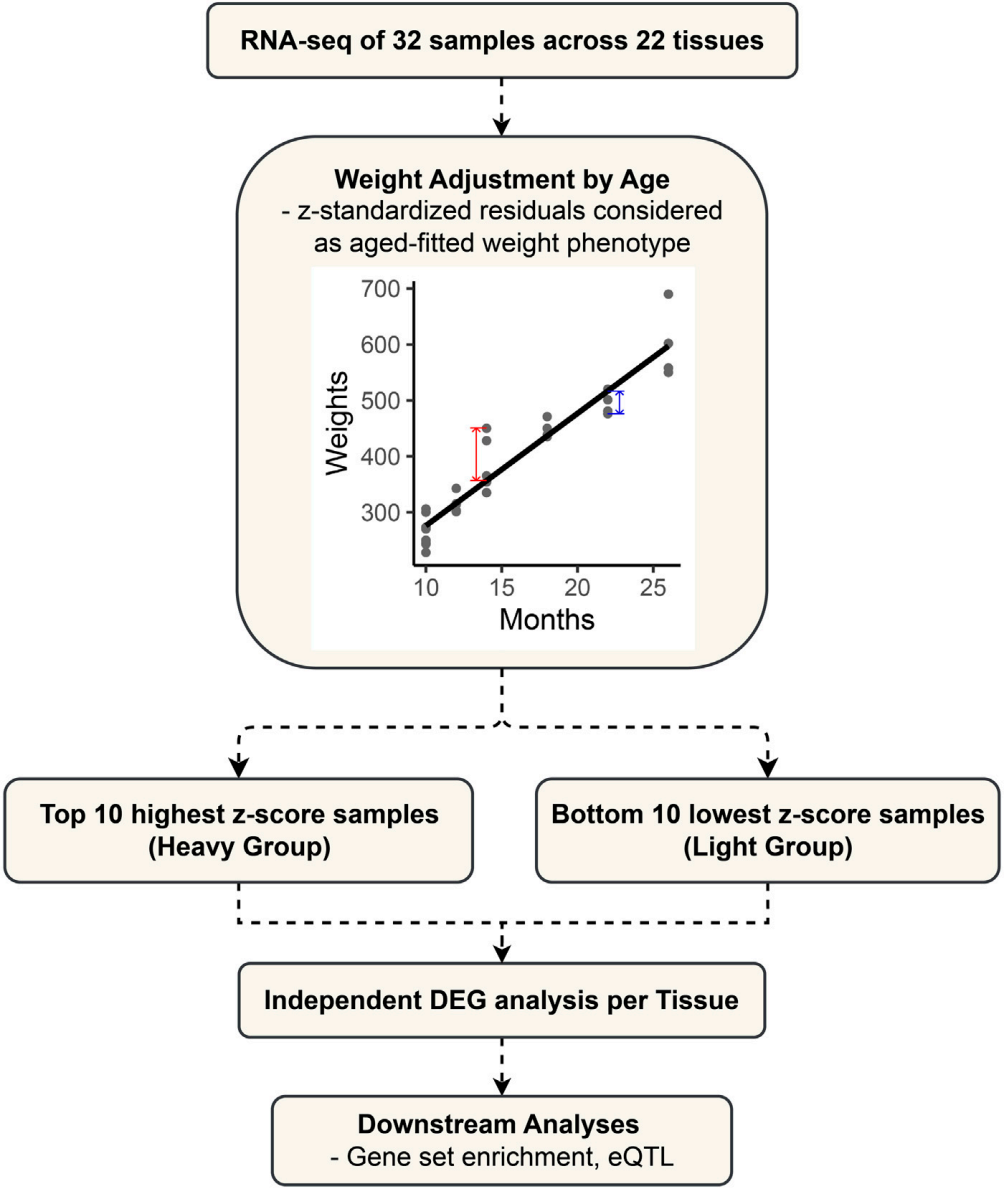
Flowchart outlining the study design and analytical approach for differential expression gene (DEG) analysis in cattle.

The 32 samples varied in age, as detailed in Supplementary Table S1. For an accurate analysis, the weight phenotype was adjusted for the effect of age (Figure 1). We adjusted the weight phenotypes for age by simple linear regression and standardized the residuals to z-scores by using the “lm” function in the R (version 4.2.2) software (Ihaka and Gentleman, 1996). The heavy group consists of the top 10 samples with the highest z-scores after age fitting, while the light group comprises the bottom 10 samples. They represent groups at the ends of a continuum in the age-adjusted weight phenotype. For each tissue, the transcriptomic comparison between the two defined groups was performed to identify differentially expressed genes.

### Principal component analysis for genetic similarity and pattern identification

To ensure the independence of the samples and to detect any potential correlations resulting from genetic or environmental influences, we performed principal component analysis (PCA). The PCA was executed using the genome-wide complex trait analysis tool (GCTA64) (Yang et al., 2011), utilizing variant call format (VCF) files obtained from RNA sequencing data of BLO (Granato et al., 2018). The VCF files were first converted to Plink format with Plink software for compatibility with the GCTA64 tool. Subsequent analyses were restricted to autosomal chromosomes to avoid confounding factors associated with sex chromosomes. The PCA was computed using the “--pca 21” option to extract the first 21 principal components. The principal components (PC1 and PC2) were adjusted as covariates in the differential gene expression (DEG) analysis to mitigate potential confounding effects.

### Differential gene expression analysis and inter-tissue correlation analysis

In the RNA-seq process, the number of reads of each gene was calculated using the FeatureCount (version 2.0.1) program (Liao et al., 2014) for the bam file generated after the STAR process (Dobin et al., 2013). Read counts were converted to counts per million (CPM), and genes with maximum CPM values less than 1 in the samples were removed. When specifying the model to be fitted, the values of PC1 and PC2 were applied as covariates to control for external factors. Differentially expressed genes (DEGs) were identified using the limma (version 3.52.4) -voom package (Law et al., 2014) of R software (Ihaka and Gentleman, 1996), and 10 samples with high z-scores were grouped as cases (= heavy group), and 10 samples with low z-scores were grouped as controls (= light group) for comparative analysis. DEGs were identified based on a *p*-value < 0.05 and | Log_2_FC | > 1. In the reference genome ARS-UCD1.2 obtained with Ensembl’s BioMart tool (https://asia.ensembl.org/index.html), overlapping analysis was performed using the Ensembl Gene ID to identify candidate DEGs.

The comprehensive assessment of gene expression correlations across all tissues was conducted using t-values derived from the limma-voom package, which was employed in the DEG analysis. To test the correlation between tissues, we utilized the Pearson coefficient test. Using the “corrplot (version 0.92)” function in R package, we distinguished and visualized three primary clusters through the hierarchical clustering algorithm (hclust).

### *cis*-eQTL analysis

Following the correlation analysis, which identified three adipose tissues [ABF (*n* = 342), BFT (*n* = 195), KIF (*n* = 160)] as significant clusters, we proceeded with an eQTL analysis aimed at uncovering variants that influence the regulatory mechanisms of gene expression for DEGs within these specified tissues. First, the previously generated vcf file, which only included SNP calling, was converted into PLINK format using VCFtools (version 0.1.13) (Danecek et al., 2011) and PLINK (version 1.90b6.24) (Purcell et al., 2007). The phenotypic dataset was represented by the TPM expression levels for each sample across all identified DEGs within the three examined tissues. Genome-wide efficient mixed model association (GEMMA) (version 0.98.5) (Zhou and Stephens, 2012) was utilized to perform association tests between the expression levels of DEGs and genotypes. Our selection criteria for candidate *cis*-eQTL variants entailed choosing SNPs that were not only on the same chromosome as their corresponding gene but also within a proximity of 500 Kb to the gene’s transcription start or polyadenylation site, with an emphasis on those exhibiting a *p*-value below 5e-05. It was run on each gene separately to identify variants with a minimum minor allele frequency (MAF) of 5%.

Next, we aimed to examine the linkage disequilibrium (LD) relationships of surrounding SNPs with the candidate variant that satisfied both the *p*-value and MAF criteria. To observe the LD relationships with a larger number of SNPs, we utilized variant data generated up to the GATK variant calling stage using RNA-seq data. The vcf file processed through the GATK pipeline was then converted to PLINK format using VCFtools and PLINK. We assessed LD relationships within a 250 Kb window on both sides of the significant SNP of interest and retrieved all reported pairs. The results were visualized using bar plots to illustrate the LD relationships between the candidate variant and other SNPs.

### Gene set enrichment analysis and visualization

DAVID v6.8 (https://david.ncifcrf.gov/) tool Field (Huang et al., 2007) was used for functional annotation and enrichment analysis of the DEG list. A *p*-value of 0.05 was used as the criterion for statistical significance. The gene set of genes reported in the GWAS catalog was identified using the GENE2FUNC process of the FUMA GWAS (https://fuma.ctglab.nl/) Field (Watanabe et al., 2017). For both tools, a *p*-value of 0.05 was used as the criterion for statistical significance.

## Results and discussion

### Experimental design and data quantification

We obtained the RNA-seq data for 22 tissue samples from 32 castrated Hanwoo cattle of different ages and weights (Materials and Methods) from the same farm managed by the Hanwoo Research Institute. Of the 32 samples, 3 samples (Sample ID: 192018, 192031, 202012) did not have sequencing data for some tissues, so the data were generated only with the tissues that had complete sequencing data (Supplementary Table S2). Statistical data regarding sequence quality and alignment information have been compiled and summarized for three adipose tissues in Supplementary Table S3. The 32 cattle were of different ages and had different body weights for each age (Supplementary Table S1). Our data show a high correlation between age and weight, and we confirmed that age explains a significant portion of the variation in body weight through the scatter plot (*R* ^2^ = 0.9075, *p*-value < 2.2E-16) (Supplementary Figure S1). Our goal was to detect differentially expressed genes (DEGs) that affect body weight and strictly control for other environmental factors. Body weight as a phenotype was adjusted considering age through simple regression analysis, and residuals were standardized as the z-score to control for the age factor (Supplementary Table S1).

In addition, we sought to control the genetic architecture and external influences on body weight among Hanwoo cattle. To achieve this, we evaluated the sample independence and potential correlations due to genetic or extrinsic factors. The analysis revealed that the first principal component (PC1) accounted for 7.0% of the variance in the data, while the second principal component (PC2) explained 5.6% (Supplementary Figure S2A). Although it seemed to form a cluster, it was confirmed that samples of similar weight or similar age were not grouped. We used a scatter plot to examine the causal or correlational relationship between PC1 and PC2 and the sample’s age, weight, and z-scores (Supplementary Figure S2B) (Kahng et al., 1998). All scatter plots showed apparent linear relationships, but PC1 × months, PC1 × body weights, PC1 × z-score, PC2 × months, and PC2 × body weights demonstrated non-significant correlations with *p*-values greater than 0.05. The scatter plot of the PC2 × z-score was considered statistically significant with a *p*-value of 0.03, but it exhibited a weak correlation with an *R* ^2^ value of 0.16. These results suggest that the formed clusters are not strongly associated with age or weight, and there are no genetically related individuals. However, it also demonstrates the necessity to consider PC1 and PC2 to obtain robust evidence of genetic mechanisms underlying body weight regulation.

### Analysis of differentially expressed candidate genes involved in body weight in each tissue

To carefully detect differential gene expression in relation to weight, we defined the heavy (case) and light (control) groups which correspond to the top and bottom 10 individuals according to the age-fitted body weight z-scores, respectively. The transcriptomic comparison between groups was performed for each tissue independently while adjusting for the covariates PC1 and PC2 (Supplementary Table S4). By comparing the control and case groups in each of the 22 tissues, we found a maximum of 602 DEGs in the ILE and a minimum of 17 DEGs in the Tenderloin (TEN) (Supplementary Table S5) (Zhou et al., 2019). The top 1 genes that were significantly identified in each tissue, *Zinc Finger Protein 385A (ZNF385A), EGF Containing Fibulin Extracellular Matrix Protein (EFEMP1), Testis And Ovary Specific TOPAZ 1 (TOPAZ1), Cytochrome P450 Family 1 Subfamily A Member 1 (CYP1A1), BARX Homeobox 1 (BARX1), FosB Proto-Oncogene (FOSB), ADAMTS like 3 (ADAMTSL3), Ubiquitin D (UBD), Solute carrier family 6 member (SLC6A14)* are known to be associated with adipogenesis, adipocyte proliferation, height, body mass index (BMI), lipid metabolism, weight and obesity-related functions (Table 1) (Suviolahti et al., 2003; Safran et al., 2010; DuBois et al., 2012; Welter et al., 2014; Skrypnik et al., 2017; Zhao et al., 2018; Rask-Andersen et al., 2019; Wang et al., 2020; Xiao et al., 2020). The genes identified that genes contributing to weight-related functions are not exclusively expressed in adipose tissue but also in other tissues such as the heart (HEA), kidney (KID), liver (LIV), sirloin (LOM), lung (LUN), omasum (OMA), rectum (REC), and reticulum (RET). This suggests a broader biological involvement of these genes across various tissue types in the regulation of body weight.

**TABLE 1:** Differentially expressed genes (DEGs) Top 1 by tissue.

Tissue	Gene	Gene name	Log2FC	*p*-value	Function & association
ABF	*NFATC1*	nuclear factor of activated T cells 1	1.107661292	2.05E-04	Estimated glomerular filtration rate in diabetes, Estimated glomerular filtration rate in diabetes, Estimated glomerular filtration rate in non-diabetics
ABO	*C1H3orf52*	chromosome 1 C3orf52 homolog	−1.22000226	2.26E-04	-
BFT	*ZNF385A*	Zinc Finger Protein 385A	1.030168135	2.37E-04	Adipogenesis through 3′-UTR binding and translational regulation of CEBPA mRNA, Body height, BMI-adjusted waist circumference
BLO	*BTBD16*	BTB Domain Containing 16	−3.192387309	1.42E-04	Bipolar disorder disease
CEC	*SCGB1D*	secretoglobin, family 1D	2.761929305	2.69E-04	-
COL	*REG3G*	Regenerating Family Member 3 Gamma	5.174855604	3.47E-04	Regenerating islet-derived protein 3-gamma levels
DUO	*BTNL9*	Butyrophilin Like 9	1.219409464	3.32E-03	Blood protein levels
HEA	*EFEMP1*	EGF Containing Fibulin Extracellular Matrix Protein 1	−1.19589485	1.41E-04	Body height, Body fat distribution, Body weight, Body mass index
ILE	*CDCA7*	Cell Division Cycle Associated 7	−1.17600901	9.35E-06	MYC-mediated cell transformation and apoptosis
JEJ	*IL21*	Interleukin 21	−2.137673447	2.05E-04	Cytokines with immunomodulatory activity
KID	*TOPAZ1*	Testis And Ovary Specific TOPAZ 1	1.771979303	3.66E-03	Body mass index, Sperm development and sperm cell division
KIF	*CD300H*	CD300H Molecule (Gene/Pseudogene)	3.114638566	2.35E-04	Involved in innate immunity and autoimmune response
LIV	*CYP1A1*	Cytochrome P450 Family 1 Subfamily A Member 1	−1.068010846	1.34E-04	Involved in the metabolism of various endogenous substrates including fatty acids, steroid hormones and vitamins
LOM	*BARX1*	BARX Homeobox 1	1.52242117	2.13E-03	Body mass index, Inhibits endoderm Wnt activity
LUN	*FOSB*	FosB Proto-Oncogene, AP-1 Transcription Factor Subunit	−1.71298643	2.74E-03	Coexistence of osteoporosis, colon cancer and obesity
OMA	*ADAMTSL3*	ADAMTS Like 3	1.038905769	4.64E-05	Body fat distribution, Body fat percentage, Abdominal adipose tissue volumes, Type 2 diabetes, Weight
REC	*UBD*	Ubiquitin D	−1.929973168	6.09E-4	Inflammation, apoptosis and tumorigenesis, Adipogenesis and proliferation
RET	*SLC6A14*	Solute Carrier Family 6 Member 14	−1.243818073	9.84E-04	Mutations in this gene are associated with X-linked obesity
RMP	*CASQ2*	Calsequestrin 2	−1.278916229	1.55E-03	Serves as an internal calcium store in muscle
RUM	*SH2D1A*	SH2 Domain Containing 1A	1.240464379	1.13E-04	Inhibitors of transmembrane proteins
SPL	*OR5E1*	Olfactory receptor family 5 subfamily E member 1	−2.866277868	5.28E-03	-
TEN	*ALB*	Albumin	−3.212922518	1.05E-03	Control of colloidal osmotic pressure in the blood

In addition, 381 genes were differentially expressed in two or more tissues (Supplementary Table S6). Notably, the *RGS2* and the *UBD* exhibited differential expression in multiple tissues. The *Regulator Of G Protein Signaling 2 (RGS2)* showed significant differential expression in 9 tissues (Abomasum (ABO), BLO, Colon (COL), ILE, Sirloin (LOM), Omasum (OMA), Rectum (REC), Spleen (SPL) and TEN), and the *UBD* was differentially expressed in 7 tissues (COL, Jejunum (JEJ), Liver (LIV), LOM, OMA, REC, Rumen (RUM)). It has been reported that the loss of *RGS2* is advantageous for glucose production but disadvantageous for glycogen and lipid production, contributing to a lean phenotype with a lower body weight (Nunn et al., 2011). This has previously been demonstrated to emphasize the importance of *RGS2* in obesity control and insulin sensitivity because it is involved in adipocyte differentiation, and impaired adipocyte differentiation can contribute to a lower body weight phenotype. The downregulation of the *UBD* has been reported in previous studies to partially inhibit adipogenesis of subcutaneous adipocyte precursor cells within pig muscles and to suppress cell proliferation, indicating its essential role in the differentiation of adipocyte precursor cells (Zhao et al., 2018). This suggests that in order to understand the mechanisms by which differentially expressed genes (DEGs) in non-adipose tissues influence body weight, additional analyses are also necessary.

### Correlation of gene expression patterns between tissues

**FIGURE 2:**
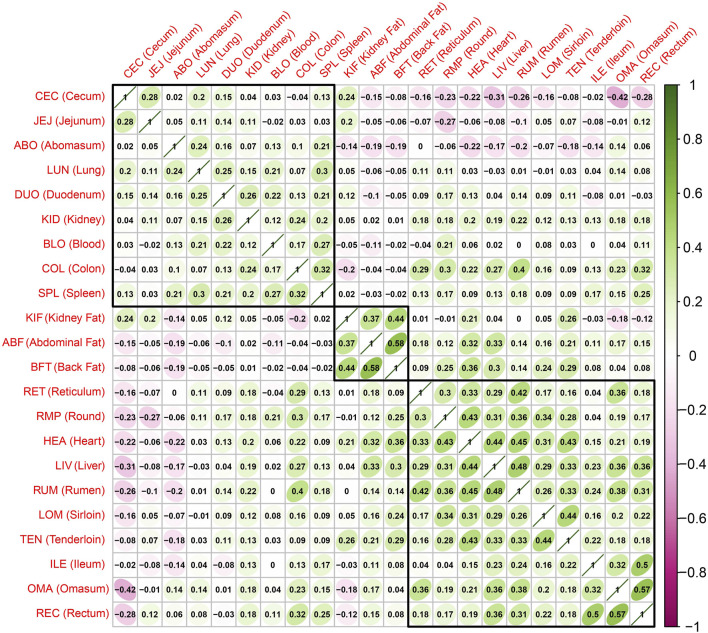
Correlations and hierarchical clustering between tissues based on the t value representing the difference in expression between the case group (sample with a high z-score) and the control group (sample with a low z-score). The closer the correlation value is to 1, the higher the correlation.

To examine the overall relationship of gene expression patterns across the entire tissue, we generated a correlation plot using the T-values from the Limma-Voom analysis, representing the expression differences between the case and control groups (Figure 2). We used a hierarchical clustering algorithm (hclust) to generate correlation plots by dividing into three major clusters to view the clustering of tissues with similar gene expression patterns. The highest positive correlation coefficient observed (correlation coefficient = 0.58) was between the Abdominal Fat (ABF) and Back Fat (BFT), and the highest negative correlation coefficient (=−0.42) was between the OMA and Cecum (CEC). One of the three clusters was identified to consist exclusively of adipose tissue, including ABF, BFT and kidney fat (KIF). This result indicates a similarity in gene expression patterns among adipose tissues, and we specifically focused on adipose tissue as the primary tissue for further investigation.

### Functional annotation of adipose tissue DEGs

**FIGURE 3:**
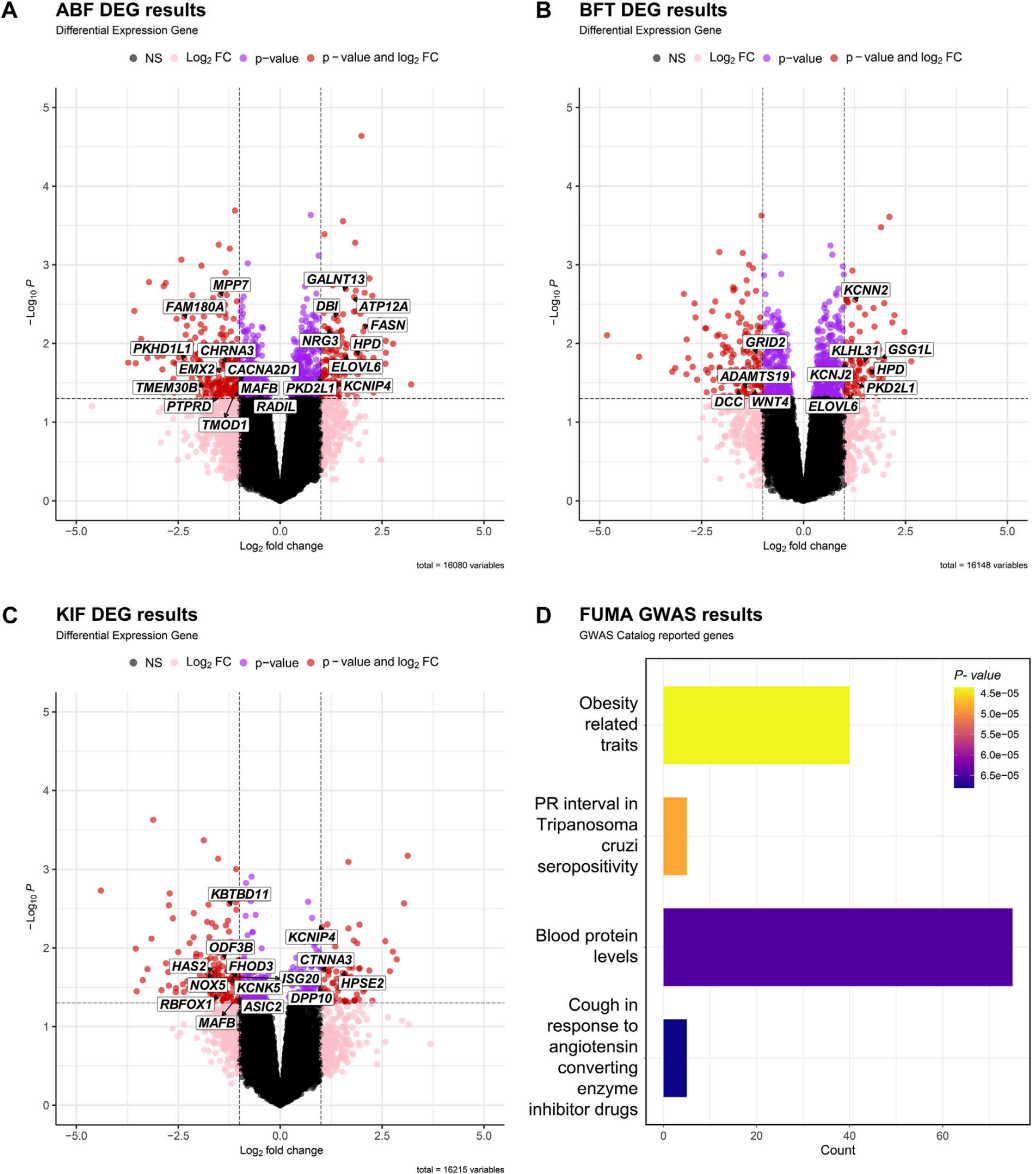
Volcano plot of DEGs in adipose tissue [**(A)** ABF, **(B)** BFT, **(C)** KIF]. The X-axis is Log_2_FC, the Y-axis is -Log_10_ *p*- value, and the cutoff criterion is *p*-value < 0.05, |Log_2_FC| > 1. Black dots are points that do not satisfy both the *p*-value and the Log_2_FC FC criterion, pink dots are points that satisfy only the Log_2_FC criterion, purple dots are points that satisfy only the *p*-value criterion, and red dots are differentially expressed genes (DEGs) that satisfy both *p*-value and Log_2_FC criteria. We displayed the genes associated with obesity-related traits based on the results of **(D)**. **(D)** Table showing the gene set reported in the GWAS Catalog based on the results of FUMA GWAS using candidate genes from the three tissues.

Figures 3A–C depict the differential expression patterns of all genes in three adipose tissues (ABF, BFT and KIF) and showcase the DEGs that were significantly selected in two or more adipose tissues. Figure 3D depicts the differential expression profiles and related enrichment analysis of significant DEGs in the three adipose tissues using the Gene Ontology (GO) (Consortium, 2004), conducted through the FUMA GWAS (Watanabe et al., 2017). The gene set enrichment analysis mapped 40 genes to the obesity-related traits (*p*-value < 0.05) among the GO terms (Figure 3D).

In the GO analysis of adipose tissue using DAVID (Huang et al., 2007), the genes were examined separately as upregulated genes and downregulated genes (Table 2). The downregulated genes, including those involved in signal transduction, organ or limb morphogenesis, bone cell differentiation or development, various metabolic biosynthetic processes, and other related biological processes (BP), were enriched with 41, 24, and 7 significant GO terms in the ABF, BFT, KIF tissues, respectively. The upregulated genes, primarily involved in metabolism, fatty acid metabolism, lipid metabolism, glutathione, and other metabolic processes, were enriched with 18, 3, and 2 significant GO terms in the ABF, BFT and KIF tissues, respectively. This indicates that most of these are associated with metabolic processes such as fatty acid and lipid metabolism and signal transduction.

**TABLE 2:** Significant gene set enrichment analysis in 3 adipose tissue (ABF, BFT, KIF) DEGs.

Tissue	Regulation	Pathways	Term	*p*-value
ABF	Downregulated	GO-Biological Pathways	Positive regulation of mesenchymal cell proliferation	3.07E-05
			Mesenchyme migration	1.19E-03
			Animal organ morphogenesis	1.62E-03
			Proximal/distal pattern formation	2.00E-03
			Positive regulation of gene expression	2.29E-03
			Embryonic limb morphogenesis	2.86E-03
			Positive regulation of canonical Wnt signaling pathway	3.54E-03
			Embryonic forelimb morphogenesis	3.93E-03
			Positive regulation of smoothened signaling pathway	4.33E-03
			Extracellular matrix organization	5.15E-03
			Osteoblast differentiation	5.18E-03
			Lymphangiogenesis	6.26E-03
			Regulation of heart contraction	6.26E-03
			Cartilage development	7.25E-03
			Chondrocyte differentiation	8.43E-03
			Embryonic digestive tract development	8.75E-03
			Positive regulation of endothelial cell migration	1.04E-02
			Inner ear morphogenesis	1.11E-02
			Negative regulation of osteoblast differentiation	1.19E-02
			Collagen fibril organization	1.26E-02
			Positive regulation of protein kinase B signaling	1.29E-02
			Intermediate filament cytoskeleton organization	1.48E-02
			Intermediate filament organization	1.84E-02
			Oogenesis	1.84E-02
			Positive regulation of cell proliferation	2.52E-02
			Wnt signaling pathway	2.57E-02
			Positive regulation of MAPK cascade	2.74E-02
			Lung development	2.87E-02
			Positive regulation of vascular endothelial growth factor production	3.08E-02
			Ureter maturation	3.28E-02
			Vascular smooth muscle contraction	3.28E-02
			Signal transduction	3.29E-02
			Positive regulation of axonogenesis	3.32E-02
			Eye development	4.32E-02
			Zymogen activation	4.32E-02
			Regulation of Ras protein signal transduction	4.35E-02
			Negative regulation of inflammatory response	4.55E-02
			Inner ear development	4.58E-02
			Negative regulation of Wnt signaling pathway	4.58E-02
			Brain development	4.76E-02
			Regulation of heart rate by cardiac conduction	4.85E-02
	Upregulated	GO-Biological Pathways	Glutathione metabolic process	1.80E-05
			ATP synthesis coupled electron transport	6.75E-04
			Mitochondrial electron transport, NADH to ubiquinone	8.85E-04
			Fatty acid biosynthetic process	2.01E-03
			Phospholipid biosynthetic process	3.40E-03
			Unsaturated fatty acid biosynthetic process	4.54E-03
			Mitochondrial respiratory chain complex I assembly	8.27E-03
			Response to stilbenoid	1.35E-02
			Regulation of phospholipid biosynthetic process	1.35E-02
			Response to glucose	1.87E-02
			Tryptophan transport	2.03E-02
			Proteolysis	3.04E-02
			Regulation of cytokine production	3.16E-02
			Protein homotetramerization	3.90E-02
			Negative regulation by host of viral process	4.01E-02
			Glycerol-3-phosphate metabolic process	4.01E-02
			Fatty acid elongation, polyunsaturated fatty acid	4.66E-02
			Lung lobe morphogenesis	4.66E-02
BFT	Downregulated	GO-Biological Pathways	Protein urmylation	2.25E-02
			tRNA wobble position uridine thiolation	2.80E-02
			Lung vasculature development	2.80E-02
			Axon guidance	4.23E-02
			Extracellular matrix organization	4.78E-02
			Tyrosine phosphorylation of STAT protein	4.99E-02
			Digestive tract morphogenesis	4.99E-02
	Upregulated		Glutathione metabolic process	3.00E-05
			CDP-diacylglycerol biosynthetic process	2.94E-02
			Axonemal dynein complex assembly	4.73E-02
KIF	Downregulated	GO-Biological Pathways	Superoxide anion generation	1.93E-06
			Extracellular matrix assembly	1.59E-03
			Thyroid hormone generation	2.24E-03
			Stabilization of membrane potential	3.85E-03
			Negative regulation of osteoclast differentiation	4.81E-03
			Positive regulation of apoptotic cell clearance	1.10E-02
			Regulation of thyroid hormone generation	1.10E-02
			Defense response to Gram-positive bacterium	1.38E-02
			Proteolysis	1.52E-02
			Melatonin biosynthetic process	1.64E-02
			Extracellular polysaccharide biosynthetic process	1.64E-02
			Positive regulation of hydrogen peroxide biosynthetic process	1.64E-02
			Negative regulation of BMP signaling pathway	2.13E-02
			Cell adhesion	2.21E-02
			Hyaluronan biosynthetic process	2.72E-02
			Hydrogen peroxide metabolic process	2.72E-02
			Ossification	2.86E-02
			Hydrogen peroxide biosynthetic process	3.25E-02
			Positive regulation of platelet aggregation	3.25E-02
			Response to light stimulus	3.78E-02
			Bone trabecula formation	3.78E-02
			Respiratory burst	4.31E-02
			Positive regulation of phosphatidylinositol 3-kinase signaling	4.70E-02
			Embryonic eye morphogenesis	4.84E-02
	Upregulated	GO-Biological Pathways	Regulation of potassium ion transmembrane transport	1.40E-04
			Regulation of membrane potential	1.29E-02

### Expression comparison of fatty acid metabolism

**FIGURE 4:**
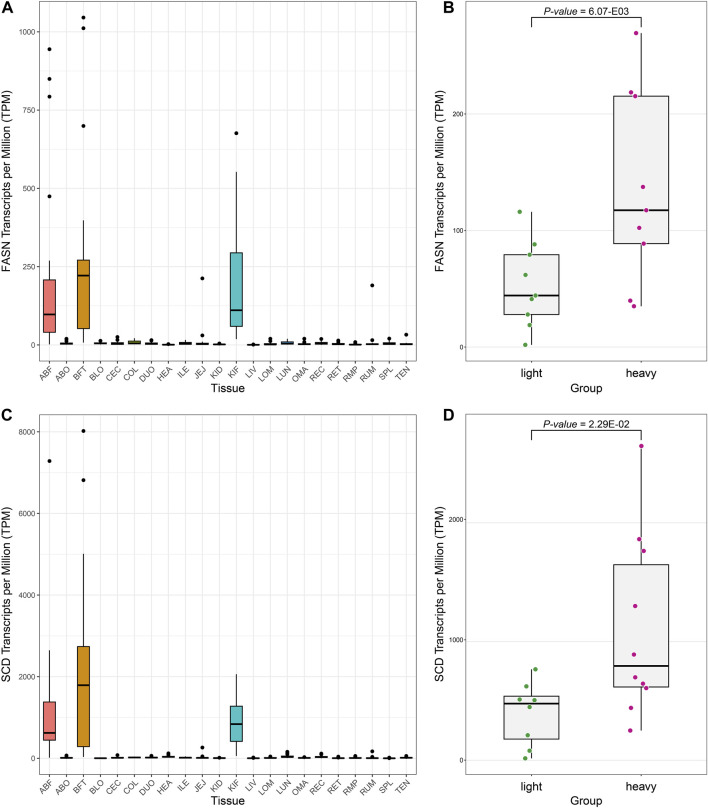
Boxplot to confirm the expression level of representative genes (*FASN, SCD*) related to fat metabolism. **(A)** The expression pattern of the *FASN* across all tissues. **(B)** Boxplot showing the differential expression pattern of *FASN* in ABF tissue based on groups (z-scores of top and bottom 10 samples). **(C)** Expression pattern of the *SCD* across all tissues. **(D)** Boxplot showing the differential expression pattern of the *SCD* gene in ABF tissue based on groups.

We discovered through gene set enrichment analysis that many DEGs are associated with metabolic processes such as fatty acid and lipid metabolism in adipose tissues. We examined the expression levels of *Fatty acid synthase (FASN)* and *Stearoyl-CoA desaturase (SCD)*, DEGs involved in well-known fatty acid metabolism according to the literature, in all tissues (Figure 4) (Zhang et al., 2018). We confirmed that the *FASN* and *SCD* are expressed significantly more in the adipose tissues than in the other tissues. *FASN* is a complex homodimeric enzyme that regulates the *de novo* synthesis of long-chain fatty acids in mammals (Chakravarty et al., 2004). It catalyzes the formation of fatty acids with a 16-carbon atom length from acetyl-CoA and malonyl-CoA (Chakravarty et al., 2004). The expression product of the *FASN* is involved in lipid metabolism, and it is known to participate in fat accumulation and fatty acid composition in pigs and cattle (Grzes et al., 2016; Raza et al., 2018). The *SCD* functions as an enzyme in mammalian adipocytes, converting saturated fatty acids into monounsaturated fatty acids (MUFAs) (Taniguchi et al., 2004). The conversion of saturated fatty acids to MUFAs by the *SCD* enzyme has a role in signal transduction, cell differentiation, and cell apoptosis. It can influence the development of certain tumor mutations (Dobrzyn and Ntambi, 2004). Considering the various roles of these MUFAs, changes in *SCD* activity in mammals can potentially impact key physiological variables such as differentiation, insulin sensitivity, metabolic rate, obesity, atherosclerosis, and cancer (Dobrzyn and Ntambi, 2004). The *SCD* is an important metabolic control point in weight regulation. One study identified it as one of the genes exerting the greatest influence on intramuscular fat content and fatty acid composition in Angus cattle through a Genome-wide association study (GWAS) (Dobrzyn and Ntambi, 2004; Ros-Freixedes et al., 2016).

### The candidate *cis*-eQTL variant regulating the expression level of *TRIM31*

In the adipose tissues of ABF, BFT, and KIF, expression quantitative trait loci (eQTL) analysis was conducted on 697 genes identified as differentially expressed, which yielded three SNPs meeting the significance threshold of *p*-value < 5e-05 and possessing a minor allele frequency (MAF) greater than 5% (Supplementary Table S7). The three SNPs that met both the *p*-value and MAF criteria were exclusively discovered in the ABF tissue; no SNPs in the BFT and KIF tissues satisfied these conditions. One SNP was located within the *UBD* and emerged as a significant variant influencing the expression levels of *Tripartite Motif Containing 31* (*TRIM31*) (chr23:29119138, *p*-value = 8.86e-06, MAF = 19.4%). The other two SNPs were positioned near the *Adenosine Deaminase RNA Specific* (*ADAR*) and identified as significant variants affecting the expression of the *Tudor Domain Containing 10* (*TDRD10*) (chr3:15995172, *p*-value = 1.31e-05, MAF = 5%; chr3:15995183, *p*-value = 1.31e-05, MAF = 5%). *TRIM31* has been identified as a “Janus-faced” regulator of innate immune responses, facilitating signal transduction through target substrate degradation or ubiquitin modification (Xu et al., 2022). Furthermore, in some studies, functional impairment (knockout) of *TRIM31* has been shown to significantly increase body weight, fasting blood glucose levels, and fasting insulin levels induced by a high-fat diet (HFD), suggesting that reduced expression of *TRIM31* can contribute to weight gain (Xu et al., 2022). Due to the lack of literature supporting the involvement of *TDRD10* in weight regulation, the SNP (chr23:29119138) within *UBD* that regulates the expression of *TRIM31* was selected as the most prominent candidate *cis*-eQTL variant associated with weight control.

**FIGURE 5:**
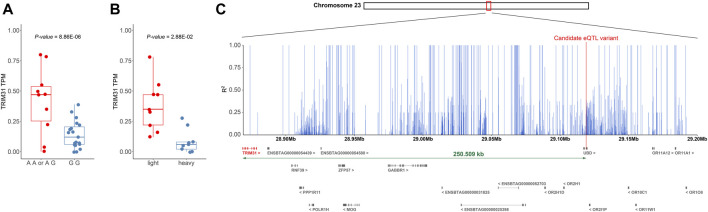
**(A)** Distance between *TRIM31* and candidate variants regulating the expression level. **(B)** Boxplot showing the change in *TRIM31* expression level according to the genotype of the candidate *cis*-eQTL variant (chr23:29119138) that affects *TRIM31* expression. **(A, B)** Additionally, outliers were excluded during the visualization process in creating the boxplot. **(C)** Bar plot depicting the LD values between the candidate *cis*-eQTL variant and surrounding SNPs within 250 Kb. The figure displays the range chr 23:28,868,629-29,200,000. A plot of the entire 500 Kb region is provided in Supplementary Figure S4. The x-axis represents genetic coordinates while the y-axis indicates the LD values between the candidate variant and SNPs. Differentially expressed gene *TRIM31* and the candidate *cis*-eQTL variant are highlighted in red.

We sought to determine whether the genotype of the candidate cis-eQTL variant (chr23:29119138) is associated with the regulation of *TRIM31* expression and body weight control. Samples with the GG genotype at chr23:29119138 showed lower expression levels of *TRIM31* compared to samples carrying the alternative allele A (*p*-value = 8.86e-06) (Figure 5A). Upon comparing *TRIM31* expression levels between the heavy and light groups, the light group exhibited a significantly higher level of *TRIM31* expression than the heavy group (Figure 5B). Although the *p*-value did not reach statistical significance, visualization of the raw body weight differences based on the genotypes at chr23:29119138 showed a trend where individuals carrying allele A exhibited a lower weight distribution (Supplementary Figure S3). These research findings, while not statistically significant, support the directionality of weight regulation associated with *TRIM31* expression previously reported in the literature (Luo et al., 2022; Xu et al., 2022). This suggests that a reduction in *TRIM31* expression may contribute to an increase in body weight, and the variant at chr23:29119138 could potentially regulate the expression of *TRIM31*.

However, the candidate *cis*-eQTL variant is located within the exon of the *UBD* gene, which has been reported in mice to be a gene upregulated by an HFD, and the deficiency of *UBD* has been associated with a reduction in body fat due to increased energy expenditure (Choi et al., 2015). Given that the *TRIM31* gene (but not the *UBD*) showed the differential expression between weight groups, the significant association of the SNP within the *UBD* gene and the expression level of *TRIM31* may have been attributable to strong linkage disequilibrium (LD) within this region. To examine this further, an LD pattern analysis was conducted within a 250 Kb range on either side of the candidate variant to assess the LD relationship with neighboring SNPs. The LD analysis revealed a high level of linkage around the candidate *cis*-eQTL variant and also confirmed a strong LD (*R* ^2^ = 1) with SNPs within the *TRIM31* (Figure 5C; Supplementary Figure S4). These findings suggest that the neighboring SNPs may be co-inherited with the candidate *cis*-eQTL variant and could be implicated in gene expression regulation, even if they are not the direct causative variants. Moreover, the candidate variant could be correlated with the causal variant due to close genetic linkage. Consequently, the candidate *cis*-eQTL variant may be closely linked to the actual causative variant or contribute to the modulation of *TRIM31* expression. This underscores the need for further investigation to elucidate the fundamental genetic mechanisms.

### Limitation

The current study’s outcomes are subject to several limitations. Weight gain over time serves as an important indicator of feed efficiency and would likely yield better performance in research outcomes. Additionally, the absence of information on environmental factors presents a limitation in completely controlling for external influences. Nevertheless, it is acknowledged that body weight is significantly influenced by both environmental and genetic factors. Particularly in humans, the heritability estimate for body weight has been reported to be between 0.7 and 0.81, while in cattle, heritability estimates vary but have been reported to range from approximately 0.3 to 0.6 (Toshniwal et al., 2008; Russo et al., 2010; Polizel et al., 2018; Rezende et al., 2022). Recognizing the weight of genetic contributions, this study meticulously incorporated principal components (PC1 and PC2) as covariates in the DEG analysis to mitigate environmental biases, thus sharpening the focus on genetic correlations with body weight.

The sample size, comprising 32 individuals, is relatively small, which restricts the ability to detect trans-eQTLs that typically exhibit smaller effect sizes compared to cis-eQTLs. In both DEG and eQTL analyses, the *p*-value significance threshold did not meet the stringent standards set by FDR adjustment, posing challenges in identifying influential genes and SNPs. This raises concerns regarding the incidence of Type I errors. The imperative for subsequent analyses with augmented datasets is clear, to yield more precise and dependable outcomes.

Despite these issues, the study leverages the convergence of DEG and eQTL analyses to enhance the reliability of the genetic associations identified. The utilization of RNA-seq data for eQTL analysis is a novel approach for Hanwoo cattle research, marking a significant contribution that paves the way for future inquiry. This research underscores the importance of continuous investigation, bolstered by broader datasets, to reinforce the preliminary findings presented.

## Conclusion

We analyzed the gene expression data from multiple tissues to identify genes and biological mechanisms at the transcriptome level that influence the weight of Hanwoo cattle. Our study has uncovered transcriptional changes associated with weight in previously overlooked tissues. We have confirmed that the candidate genes we discovered are associated with biological pathways involving various metabolic processes, such as lipid metabolism, adipogenesis, and adipocyte proliferation. Using RNA-seq data in expression quantitative trait loci (eQTL) studies enabled us to identify allele-specific gene expression easily. By integrating eQTL and differentially expressed genes (DEGs) analysis results, we have identified genomic regions that may regulate the expression of candidate genes, such as *TRIM31* and provided insights into their association with the expression levels. Of particular interest, we found that the variant regulating the expression of *TRIM31* is located within the *UBD*, which is known to regulate adipogenesis. The findings suggest that further analysis is necessary to fine-map causal *cis*-eQTL variants regulating *TRIM31*. Moreover, it emphasizes the necessity to broaden the focus and understanding of research on various tissues that can influence the weight of Hanwoo cattle and other livestock. Our study may represent a comprehensive genomic and transcriptomic portrait of livestock body weight by utilizing the RNA-seq data of many tissues and progress toward understanding the role of eQTLs in determining livestock phenotypic diversity.

## Data Availability

The datasets presented in this study can be found in online repositories. The names of the repository/repositories and accession number(s) can be found in the article/Supplementary Material.
