# Supplementary material for: Multi-tissue transcriptome analysis to identify candidate genes associated with weight regulation in Hanwoo cattle
Source: Front Genet. 2024 Jan 9;14:1304638. doi: 10.3389/fgene.2023.1304638 (PMC10803631; doi:10.3389/fgene.2023.1304638)

Supplementary Figure 1. This is a scatter plot to examine the relationship between age and weight changes, as the samples have different ages.

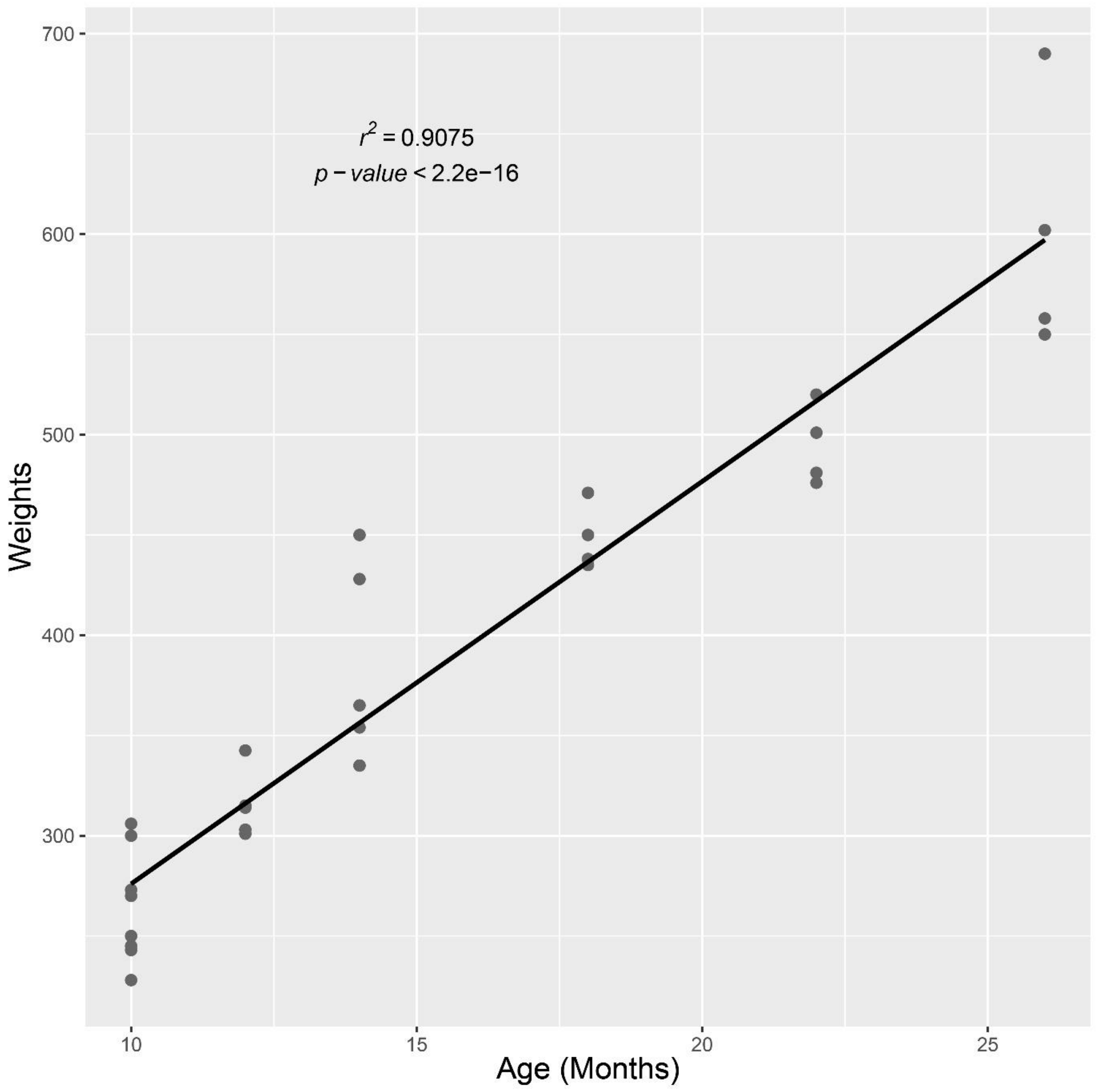

Supplementary Figure 2. (A) A PCA analysis was conducted to determine if an external factor had worked in determining the body weight of the samples. (B) Scatterplot to determine if there is a linear relationship between the similarity in DNA and the similarity in body weight between samples : PC1 and body weight, PC1 and age-adjusted z-score, PC1 and age in months, PC2 and body weight, PC2 and age-adjusted z-score, PC2 and age in months

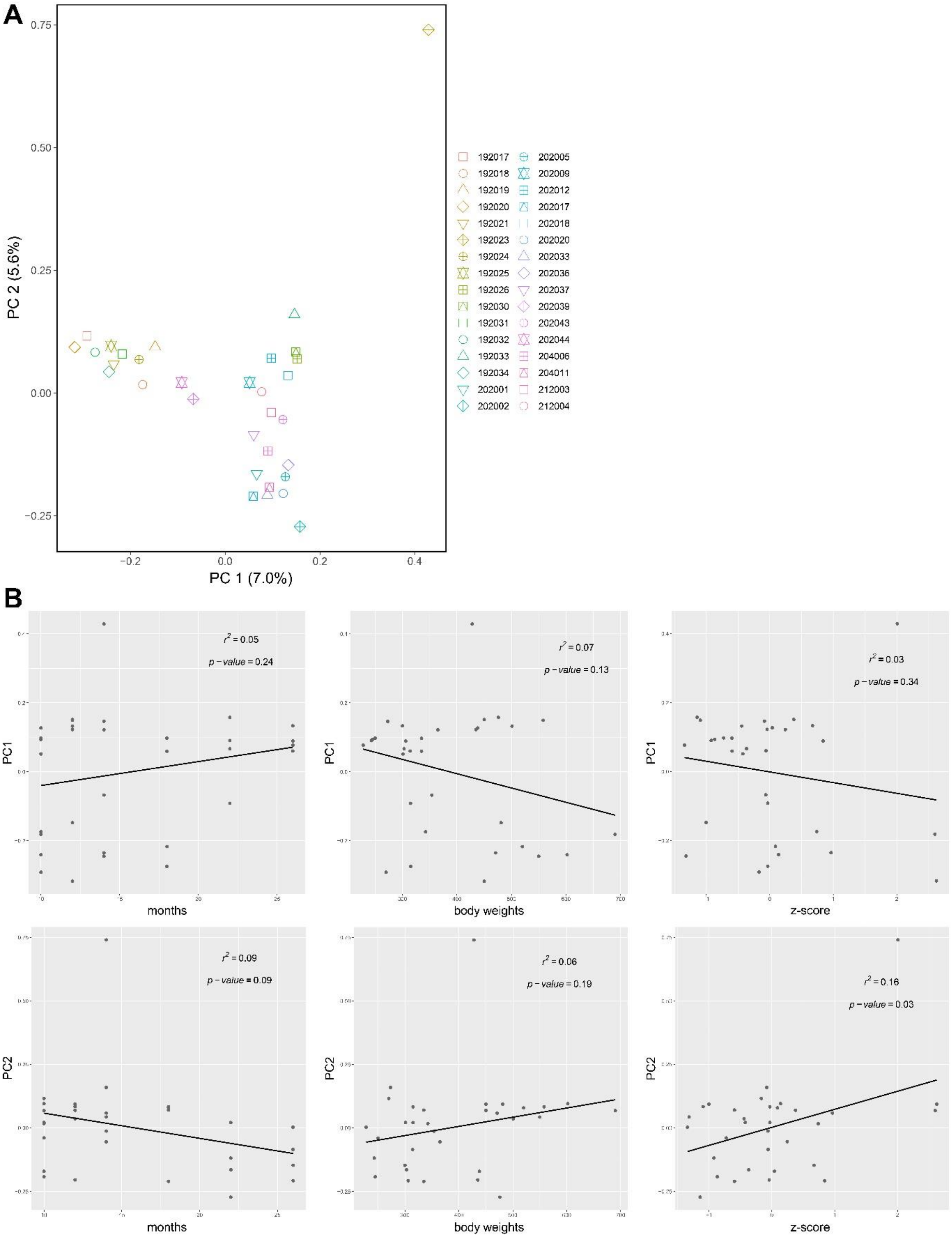

Supplementary Figure 3. Boxplot depicting body weight (z-score) changes based on the genotype of the candidate eQTL variant chr23:29119138.

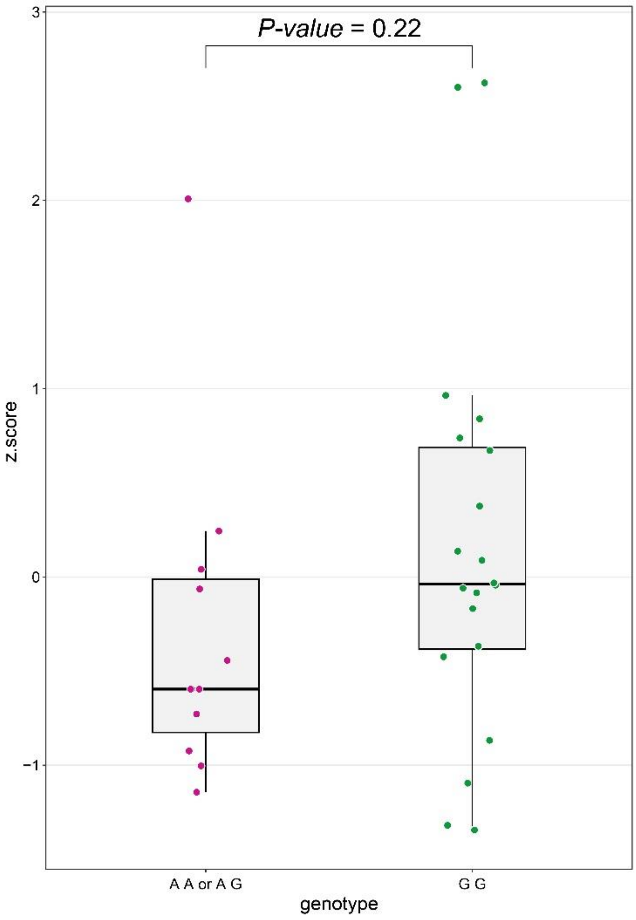

Supplementary Figure 4. Bar plot depicting the LD values between the candidate cis-eQTL variant and surrounding SNPs within 250Kb. The x-axis represents genetic coordinates while the y-axis indicates the LD values between the candidate variant and SNPs. Differentially expressed gene TRIM31 and the candidate cis-eQTL variant are highlighted in red.

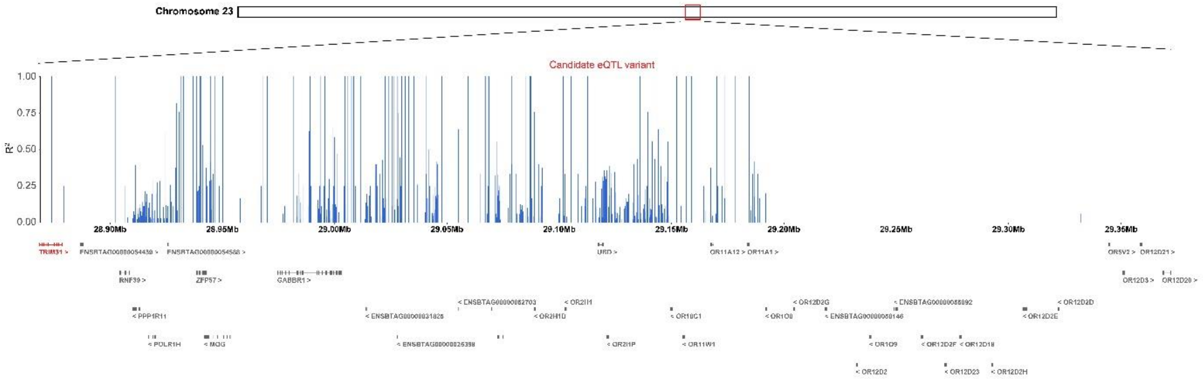

Supplement: Supplementary file 2 [file Image1.pdf]
